# Cardiac magnetic resonance versus transthoracic echocardiography for the assessment of cardiac volumes and regional function after myocardial infarction: an intrasubject comparison using simultaneous intrasubject recordings

**DOI:** 10.1186/1476-7120-7-38

**Published:** 2009-08-18

**Authors:** Blake I Gardner, Scott E Bingham, Marvin R Allen, Duane D Blatter, Jeffrey L Anderson

**Affiliations:** 1 Cardiovascular Department, Intermountain Medical Center, Intermountain Healthcare, 5121 South Cottonwood Street, Murray UT 84157, USA; 2 University of Utah School of Medicine, 30 North 1900 East Room 1C109, Salt Lake City, Utah 84132, USA; 3 Central Utah Clinic, 1055 North 500 West Suite 101, Provo, Utah 84604, USA

## Abstract

**Background:**

Although echocardiography is commonly used to evaluate cardiac function after MI, CMR may provide more accurate functional assessment but has not been adequately compared with echo. The primary study objective was to compare metrics of left ventricular volumes and global and regional function determined by cardiac magnetic resonance (CMR) and echocardiography (echo) in patients (pts) with recent myocardial infarction (MI).

**Methods:**

To compare CMR with echo, 47 consecutive patients (pts 70% male; mean age = 66 ± 11 years) with MI >6 wks previously and scheduled for imaging evaluation were studied by both echo and CMR within 60 min of each other. Readers were blinded to pt information. Pearson's correlation coefficient, paired *t*-tests, and chi-square tests were used to compare CMR and echo measures. Further comparisons were made between pts and 30 normal controls for CMR and between pts and published normal ranges for echo.

**Results:**

Measures of volume and function correlated moderately well between CMR and echo (r = 0.54 to 0.75, all p < 0.001), but large and systematic differences were noted in absolute measurements. Echo underestimated left ventricular (LV) volumes (by 69 ml for end-diastolic, 35 ml for end-systolic volume, both p < 0.001), stroke volume (by 34 ml, p < 0.001), and LV ejection fraction (LVEF) (by 4 percentage point, p = 0.02). CMR was much more sensitive to detection of segmental wall motion abnormalities (p < 0.001). CMR comparisons with normal controls confirmed an increase in LV volumes, a decrease in LVEF, and preservation of stroke volume after MI.

**Conclusion:**

This intra subject comparison after MI found large, systematic differences between CMR and echo measures of volumes, LVEF, and wall motion abnormality despite moderate inter-modality correlations, with echo underestimating each metric. CMR also provided superior detection and quantification of segmental function after MI. Serial studies of LV function in individual patients should use the same modality.

## Background

Cardiac magnetic resonance (CMR) is increasingly utilized for dynamic imaging of the heart with the expectation that it will provide more accurate and reproducible measurements of cardiac chamber dimensions, volumes, and function compared to other non-invasive imaging techniques such as echocardiography and nuclear cardiography [1-3]. This expectation arises from the superior spatial resolution and more precise border definition achieved with CMR compared with these other techniques. However, the potential of CMR to be integrated optimally into clinical practice for assessment of cardiac metrics has been limited in part by a lack of comparative information with echocardiography (echo), the long-time clinical standard for non-invasive cardiovascular imaging, in health and disease. Myocardial infarction (MI) is the most common cardiovascular disease state routinely requiring imaging assessment. Little direct comparative information is available for these 2 techniques [4]. Thus, we undertook an intra subject comparison of echo with CMR in a consecutive cohort of post-MI patients to determine correlations and systematic differences between these modalities in assessing left ventricular volumes and function.

## Methods

### Study Objectives

The main study objectives were: 1) to determine and compare metrics of left ventricular volumes and function between CMR and echo in a consecutive cohort of post-MI patients presenting for imaging evaluation of cardiac function, 2) to compare the sensitivity of the 2 modalities to assess regional wall motion (RWM) after MI, and 3) to quantitatively assess changes in these parameters after MI by a comparison of study patients with a cohort of normal volunteers.

### Study Population

Forty-seven study subjects were prospectively enrolled from a consecutive series of patients with MI occurring >6 wks previously referred for echo evaluation of cardiac function and who consented to study participation, which included undergoing the complementary CMR study within 1 hour of echo. The protocol was approved by the Western Institutional Review Board. Standard electrocardiogram (ECG) and serial cardiac biomarker analysis was used to document MI.

Each subject served as his/her own control. Predefined cardiovascular measurements were made for CMR and echo using workstation-specific methods by a seasoned observer blinded to results of the complementary imaging method. Imaging results and demographic information were entered into a study database.

Given limited information on the normal range for CMR measurements and dependence of work-station specific methodology, we determined a normal range for CMR metrics in a group of 30 local normal volunteers recruited concurrently [5]. These subjects were consenting, uncompensated adults of either sex (equally divided between men and women) of ages between 40–60 years who underwent a standard CMR functional study and who had no clinically apparent cardiovascular diseases, including hypertension or diabetes on screening history, or abnormal physical on screening examination, or significant other-organ diseases. Echocardiographic normal values were taken from the published literature.

### Cardiac Magnetic Resonance Imaging (MRI) and Echo Studies

Following axial and sagittal localizer sequences, standard cardiac 2, 3, and 4 chamber 1 cm thick long axis and short axis slices were obtained on a General Electric (GE) 1.5 Tesla magnet (EXCITE platform, version 11.0) using steady state free precession (SSFP) cine sequences (typical in-plane resolution 2.2 × 1.3 mm^2^). All images were acquired using a phased-array 8-channel cardiac coil during single breath-holds (maximum, 15 seconds) with ECG (preferred) or peripheral (finger pulse) triggering. Acquisition parameters were as follows: zoom mode, TR = 2.4 msec, TE = min full, flip angle = 45°, field of view = 35 × 35 cm, matrix = 192 × 160, NEX = 0.5, slice thickness = 8 mm with zero spacing, phases = 20, and views per segment ≤ 24 to maintain temporal resolution < 80 msec. No vasoactive agents were given between echocardiography and CMR imaging.

Echocardiographic images were obtained in the standard parasternal long and short axis and apical 4 chamber and 2 chamber views utilizing digital Vivid 7 ultrasound equipment with a combined tissue imaging 2.5 – 4.0 MHz transducer (GE, Milwaukee, WI). At least 3 cardiac cycles were captured at the left ventricular (LV) base, mid papillary muscle level, and apex for wall motion assessment. No intravenous echocardiographic contrast agent was used. Later, an expert reader obtained measurements off-line from the parasternal and apical windows blinded to patient identity, gender, and clinical data. Two dimensional (2D) echo ventricular volumes and LV ejection fraction were planimetered from the 4-chamber and 2-chamber areas using the modified Simpson's rule. All of the measurements were obtained in concordance with American Society of Echocardiography standards [6].

### Image Analysis

Off-line image post-processing was performed on a cardiac Delta workstation with ReportCard 2.0 software (GE, Waukesha, WI). Measurements followed standard CMR procedures or mimicked the approach to echo measurement where appropriate. Image grayscale was adjusted to maximize the myocardial blood pool contrast without maximal pixel intensity saturation. Manual planimetry was performed by an expert CMR technologist and independently confirmed by the CMR physician reader. (Observers were blinded to echo results.) In the short axis view, the most basal slice used for LV volume analysis excluded any LV outflow tract. Unattached papillary muscles were included within the left ventricular chamber. Volumes for CMR were determined from the stack of short axis slices using Simpson's rule and for echo using the modified, semi-Simpson's rule based on 4- and 2-chamber planimetry [1,7,8]. For both modalities, floating papillary muscles were included within the chamber volume measurements. For both modalities, wall segments were divided by consensus into 2 general categories: good/adequate and suboptimal/poor. To provide a conservative comparison of the 2 modalilties, poorly imaged segments were excluded from the comparative analyses.

Consensus quantitative results were entered prospectively into research databases, and computer-assisted manual tracings were saved for later visual comparisons. Similarly, echo image analysis was performed by experienced observers blinded to CMR study results and followed standard techniques [7-9].

Segmental wall motion analysis used a standard 17-segment model [10].

### Study Variables

Study demographic variables included subject age, sex, heart rate, weight, height, and body surface area (BSA). Cardiovascular variables included LV end-diastolic (EDV) and end-systolic (ESV) volumes and the derived variables stroke volume (SV, SV = EDV-ESV), and LV ejection fraction (EF = SV/EDV). For RWM, the LV was divided into 17 segments [11], and each visualized segment was graded on a scale of 1 to 5 as normal, hypokinetic, akinetic, or dyskinetic, or aneurysmal, respectively. Wall motion then was assessed as a summed total score, a worst segmental score, and an average RWM score, calculated as the average of all scored segments [7].

### Statistics

Results are presented as mean (standard deviation). Pearson's correlation coefficient, paired *t*-tests, and chi-square tests were used to compare intra subject and inter modality CMR and echo metrics, as appropriate. SPSS for Windows (version 14.0, SPSS Inc., Chicago, IL) was used for statistical analysis. A p value of 0.05 or less was deemed nominally significant and 0.01 or less definitely significant, given the 5 primary outcome variables.

## Results

### Subject Characteristics

50 patients were initially enrolled. Three patients were excluded from study due to a rhythm other than sinus. Thus, the primary study population included 47 patients surviving MI that occurred at least 6 weeks previously. Demographics and selected patient characteristics are shown in Table 1. Mean age averaged 66 ± 11 years; 70% of subjects were male.

**Table 1:** Baseline Characteristics of the Post-MI Patient Cohort

Metric (unit)	Frequency/Descriptive
N	47
Age: y, mean (SD)	66 (11)
Sex: % (n) male	70 (33)
Heart rate: beats/min, mean (SD)	64 (14)
Body surface area: m^2^, mean (SD)	1.96 (0.23)
Location of WMA^10 ^*:	
Anterior/Anteroseptal†, % (n)	62 (29)
Inferior†, % (n)	77 (36)
Lateral†, % (n)	45 (21)
Apical†, % (n)	68 (32)
None†, % (n)	4 (2)

*Wall motion abnormality (WMA) assessed by CMR using the standard 17 segment model with assigned coronary territories^10^†

The CMR control group included 30 healthy volunteers (15 women, 15 men) of average age 47 years (range, 40 to 60) with average BSA 1.7 sq m in women and 2.1 sq m in men. Study quality was excellent for all volunteers.

### CMR and Echo Volumetrics

**Figure 1:**
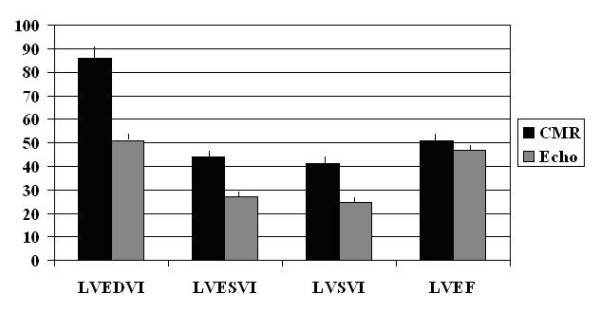
**LV Volumes (Indexed to BSA) and Function by CMR versus Echo**. Abbreviations: CMR = cardiac magnetic resonance; echo = echocardiography. Left ventricular end diastolic volume index (LVEDVI), left ventricular end systolic volume index LVESVI), left ventricular stroke volume index (LVSVI), left ventricular ejection fraction (LVEF). Volumes are indexed to body surface area (BSA). Bars represent means, given with standard error whiskers. Comparisons of LVEDVI, LVESVI, and LVSVI between CMR and echo are significant at p < 0.001, and LVEF at p = 0.02.

The volumetric measures of LVEDV, LVESV, and LVSV correlated moderately well between CMR and echo (r = 0.54 to 0.75, all p < 0.001), but large and systematic differences were noted in absolute measurements between the two (Table 2, Figure 1). Echo underestimated all 3 volumes: LVEDV by an average of 69 ml, LVESV by 35 ml, and stroke volume by 34 ml (Table 2).

**Table 2:** Summary of LV Volumes and Function by CMR versus Echo in the Post-MI cohort: Means (SD)

Metric (unit)	CMR	Echo	p-value	Correlation(r)	p-value
LV EDV (ml)	171 (62)	102 (42)	<0.001	0.701	<0.001
LV ESV (ml)	88 (47)	53 (28)	<0.001	0.746	<0.001
LV SV (ml)	83 (24)	49 (22)	<0.001	0.536	<0.001
LV EF (%)	51 (11)	47 (11)	0.02	0.672	<0.001
Worst WMS (units)	2.79 (0.88)	1.98 (0.85)	<0.001	0.692	<0.001
Total WMS/pt	26.0 (7.0)	23.0 (7.0)	0.001	0.657	<0.001
Average WMS	1.53 (0.41)	1.39 (0.42)	0.001	0.657	<0.001

**Abbreviations: **CMR = cardiac magnetic resonance; echo = echocardiography; MI = myocardial infarction. Left ventricular end diastolic volume (LVEDV), left ventricular end systolic volume (LVESV), left ventricular stroke volume (LVSV), left ventricular ejection fraction (LVEF), 17 segment wall motion score (WMS)

### Global and Segmental Function by CMR and Echo

Global LV ejection fraction correlated moderately well between the 2 modalities (r = 0.67, p < 0.001) (Table 2). However, echo underestimated LVEF compared to CMR by 4 percentage points (p = 0.02).

All 799 segments in all 47 study patients could be scored by CMR, whereas 22 (2.8%) segments (p < 0.0001) in 9 patients (19%)(p = 0.005) could not be adequately visualized to allow scoring by echo and are excluded from the comparative wall motion analyses with CMR.

In keeping with global function, segmental wall motion scores (WMS) showed moderately good correlations between CMR and echo, both for the worst WMS (r = 0.69, p < 0.001) and for total and average segmental WMS (r = 0.66, p = 0.001)(Table 2). However, echo underestimated WMS of the worst segment (by 0.81 grade, p < 0.001), as well as the average WMS and the summed WMS compared to CMR (both p < 0.001)(Table 2). Wall motion was completely normal in 15 patients (32%) assessed by echo, compared to only 2 (4%) assessed by CMR (p < 0.001), suggesting that CMR is more sensitive in distinguishing diseased from normal subjects.

### Subgroup Analysis by Degree of LV Dysfunction

To see whether differences in metrics by modality are affected by degree of LV dysfunction, comparisons were made between subgroups of patients with less than versus more than or equal to the median echo LVEF of 46%. For both modalities, LVEDV and LVESV increased in the lower LVEF subgroup, whereas LVSV was unchanged. However, inter modality differences in paired CMR versus echo LVEF and volumetrics were not significantly dependent on LVEF subgroup (i.e., mean ΔLVEF, 1.4% vs. 4.8%, p = 0.2; mean ΔLVEDV 58 ml vs. 77 ml, p = 0.15; high vs. low LVEF subgroups, respectively).

### Post-MI Patients and Normals Compared by CMR

Table 3 compares the primary volumetric and functional metrics, determined by CMR, between the post-MI patient cohort and normal volunteers, indexed to BSA. Stroke volume is preserved but is associated with moderate decreases in LVEF accompanied by moderate to large increases in LVEDVI and LVESVI

**Table 3:** Comparison of Metrics in Post-MI versus Normal Subjects by CMR Indexed to Body Surface Area*: Means (SD)

Metric (unit)	Post-MI (N = 47)	Normals (N = 30)	p-value
LV EDVI* (ml/m^2^)	86 (28)	68 (10)	0.004
LV ESVI* (ml/m^2^)	44 (22)	25 (7.0)	<0.001
LV SVI* (ml/m^2^)	41 (11)	42 (5.0)	0.72
LV EF (%)	51 (11)	65 (6.0)	<0.001

*ANOVA adjusted for sex.

**Abbreviations: **CMR = cardiac magnetic resonance. MI = myocardial infarction. Left ventricular end diastolic volume index (LVEDVI), left ventricular end systolic volume index (LVESVI), left ventricular stroke volume index (LVSVI), left ventricular ejection fraction (LVEF)

### Post-MI Patients and Normals Compared by Echo

Table 4 compares the primary volumetric and functional metrics, determined by echo, between the post-MI patient cohort and a report of normal values. LVEF is moderately decreased compared to normals. LVEDV and LVESV differences from normal values are modest and overlapping.

**Table 4:** Comparison of Metrics in Post-MI Patients versus Normal Subjects by Echo Indexed to Body Surface Area*: Means (SD)

**Metric (unit)**	**Post-MI (N = 47)**	**Normals**^12^*
LV EDVI* (ml/m^2^)	51 (19)	54.5 (9)
LV ESVI* (ml/m^2^)	27 (13)	22 (5)
LV SVI* (ml/m^2^)	25 (12)	22.5†
LV EF (%)	47 (11)	60 (6)

†Derived estimate

**Abbreviations: **MI = myocardial infarction. Echo = echocardiography. Left ventricular end diastolic volume index (LVEDVI), left ventricular end systolic volume index (LVSVI), left ventricular stroke volume index (LVESVI), left ventricular ejection fraction (LVEF)

### Indirect Comparison of Normal Ranges for CMR vs. Echo

As with the intra subject comparisons of CMR and echo in post-MI patients, an indirect comparison of the normal ranges for volunteers in our study and with a published normal range for echocardiographic metrics also suggests important differences in volumes (Table 3 vs. 4) [12].

## Discussion

### Study Overview

We performed a moderately large comparative CMR and echo imaging study of post-MI patients in which each subject served as his or her own control. We found that metrics of LV volumes and function, acquired from near simultaneous studies, while correlating moderately well, showed large and systematic inter-modality differences. Echo underestimated all primary measures compared with CMR, including LVEDV, LVESV, SV, LVEF, and, importantly, RWM abnormality. CMR also was more sensitive in distinguishing diseased from normal subjects. Of clinical importance, these differences by modality suggest that serial measurements of function should be performed using the same method, as large and spurious differences may be seen if modalities are switched. Finally, given its more accurate and reproducible determination of function, CMR should be preferred when small to moderate serial changes in these functional parameters are of clinical importance to disease management.

### Previous Work and Study Rationale

Echocardiography is a mature imaging modality [13,14] with an accepted if not extensively studied normal range for ventricular volumes [7,12,15,16]. Normal values for contemporary CMR also are now published [5,17-20]. Indirect and direct comparisons of these studies suggest that echocardiographic normal values may not accurately reflect the normal range for ventricular [4,7,19] or atrial [21] chamber measurements by CMR. A study in which each imaging modality is tested (near) simultaneously within the same subjects, and which uses carefully standardized techniques and blinded assessment, represents the most accurate and informative design to compare imaging modalities. Because coronary artery disease represents the most common reason for applying these imaging tests, we chose a typical post-MI population for a direct, intra subject comparative study of these 2 modalities.

Maciera and Royal Brompton Hospital (London) collaborators have reported on normalized left ventricular systolic and diastolic function by CMR in a moderately large study sample (n = 120) using contemporary scanner (1.5 T) and sequence (SSFP) techniques [17]. CMR analysis used a computer-based technology with blood pool thresholding to delineate the papillary muscles, which were excluded from chamber volume and included in mass measurements. As might be expected, mass measurements by this method were larger and volume measurements smaller than results obtained with the operator-interactive inclusion method used here, emphasizing the need for method-specific normal values. Alfakih also has reported normal ranges for CMR using SSFP sequences [18], but some of the same issues limit the comparison of their results with the normal values derived for the present study [5].

Normal values for cardiovascular function by MRI also were recently assessed in 800 adult participants, equally representing 4 ethnic groups, both sexes, and 4 age decades, in the multi-ethnic study of atherosclerosis [19]. As in our study, papillary muscles were included in LV volume and were excluded from LV mass calculations. Results were generally similarly to those in our population reported here for LV volumes and ejection fraction.

Little previous information is available directly comparing these two modalities in coronary artery disease. Jenkins et-al published a comparative study in 50 patients with previous MI [4]. Patients with poor echo images were excluded. Correlations of MRI versus echo for LVEDV, LVESV, and LVEF were generally similar (i.e., r = 0.61 to 0.81, p < 0.01) to those we observed (Table 2). LV volumes were slightly larger and LVEF lower in their report, indicating a somewhat sicker population. Also consistent with our observations, volumes and LVEF were under estimated by two-dimensional echo. Their study also assessed incremental change after MI over a 1-year interval, but, unlike our study, it did not assess or compare regional wall motion between modalities.

### Explanatory Mechanisms

This study did not directly determine the reasons for underestimations of volumes by echo compared with CMR. However, lower spatial (and temporal) resolution of echo, especially at far field, together with suboptimal acoustic windows in some patients with less complete visualization of wall segments, and greater "blooming" of lumen/wall boundaries with echo are disadvantages relative to CMR and likely contributors to observed differences.

### Clinical Implications

Quantitative measurements of cardiovascular anatomy and function are only of use if they are accurate and reproducible and if they can be compared with a normal, expected range of values. Incorrect interpretations, based on an incorrect comparative range of normal, large re-test variability, or spurious differences on serial testing with mixed modalities can lead to inappropriate disease assessment and management. Thus, the positive impact on clinical practice of accurate, reproducible measures of LV volumes and segmental and global function cannot be over emphasized. Despite these advantages of CMR, echocardiography has been well demonstrated by large and multicenter studies to be effective in evaluating and stratifying patients after MI with LV dysfunction. The clinical database for CMR, although considered a gold standard, is still substantially smaller. The choice of an imaging option should take cost as well as technical comparisons into account to achieve an optimal cost-effectiveness result. CMR is a moderately more expensive modality: our current Medicare facility/professional reimbursement rates for non-contrast studies are USD $679/121 (CMR) versus $412/70 (echo), with full price charges often significantly higher. Beyond this, CMR requires moderately increased acquisition and analysis times and greater equipment and facility expenditures.

### Study Strengths and Limitations

The study presents a direct comparison of imaging modalities in a primary study population sample of coronary artery disease patients, for whom imaging was clinically indicated. Only one study was obtained for each modality, so that re-test variability could not be tested. Similarly, measurements were arrived at by consensus, so that inter observer variability cannot be assessed. Other published reports provide some information on these measures [4,5,19]. Although slight differences in measurement methodology (Simpson's vs. semi-Simpson's) could contribute to results, study methods represent those most commonly used and validated in practice for each modality. Also, given close intra-modality correlations between these 2 methods, any differences due to measurement methodology are likely to be very small. Contrast echocardiography and three dimensional (3-D) echocardiography may provide more accurate LV volumetric and functional measures than standard 2D echocardiography, performed here, and may also correlate better with CMR [4]. (It should be noted that 3-D CMR also is becoming available and also may improve CMR slice selection.) However, these techniques may add time and expense to standard 2D techniques and are not fully incorporated into routine clinical practice. The study is of only moderate size and in a specific population, although size was adequate to demonstrate systematic differences in the studied metrics. Cost-effectiveness considerations also must be considered in clinical practice but are not specifically addressed by this study. Finally, the reference control group could be studied by CMR only.

## Conclusion

In a moderately large CMR versus echocardiographic comparative study in post-MI patients in which each patient served as his or her own control, metrics of LV volume and function, while moderately correlated, showed significant systematic inter modality differences, with echo underestimating all 5 primary measurement variables. CMR also was more sensitive in distinguishing wall motion abnormalities. Of clinical importance, these differences by modality suggest that serial measurements of function should be performed using the same method. Given its greater accuracy and reproducibility, CMR may be preferred when small to moderate serial changes in these metrics are clinically important.

## Competing interests

The authors declare that they have no competing interests.

## Authors' contributions

BIG participated in study planning and design, performed data analysis, and helped to draft the manuscript. SEB participated in study planning and design, took part in data acquisition, performed data analysis, and assisted with manuscript revision. MRA took part in data acquisition, performed data analysis, and assisted with manuscript revision. DDB took part in data acquisition, performed data analysis, and assisted with manuscript revision. JLA participated in study planning and design, took part in data acquisition, performed data analysis, helped to draft the manuscript, and assisted with manuscript revision. All authors read and approved the final manuscript.
